# 2-Dimensional *in vitro* culture assessment of ovarian cancer cell line using cost effective silver nanoparticles from *Macrotyloma uniflorum* seed extracts

**DOI:** 10.3389/fbioe.2022.978846

**Published:** 2022-08-16

**Authors:** Kousalya Lavudi, Venkata Satya Harika, Rekha Rani Kokkanti, Swaroopa Patchigolla, Anupriya Sinha, Srinivas Patnaik, Josthna Penchalaneni

**Affiliations:** ^1^ KIIT School of Biotechnology, KIIT University, Bhubaneswar, Odisha, India; ^2^ Department of Biotechnology, Sri Padmavati Mahila Visva Vidyalayam, Tirupati, Andhra Pradesh, India

**Keywords:** *in vitro* studies, Mitochondria membrane potential, ovarian cancer, *Macrotyloma uniflorum*, AgNPs

## Abstract

Our research focused on generating AgNPs using *Macrotyloma uniflorum* (MU) seed extracts and studied their efficacy in combating tumor growth using the 2-Dimensional method for ovarian cancer cell line-PA-1. Characterization studies including a UV-visible spectrophotometer confirmed the surface plasmon resonance peak of 436 nm. Particle size determination data validated the nanoparticle diameter of 91.8 nm. Synthesized AgNPs possess a negative charge of -28.0 mV, which was confirmed through the zeta potential study. Structural characterization studies including XRD determined the crystal phase of AgNPs at four distant peaks at 2θ (38.17, 44.36, 64.52, and 77.46) and were assigned to 111, 200, 220, and 311 planes of the FCC. FTIR studies have confirmed the presence of O-H, N-H, C=O, ethers, C-Br, and C-I groups in AgNPs respectively. DPPH study has confirmed the presence of free radicles and we observed that at 500 μg/ml concentration, 76.08% of free radicles were formed which shows their efficiency. MTT assay shows the efficacy of MU-AgNPs in reducing the cell viability. At lower concentrations of MU-AgNP, 66% viability was observed and 9% of viability was observed at higher dose. ROS production (21%) was observed using MU-AgNPs with respect to 0.45% in controls, which affirms the capacity to induce DNA damage via apoptosis. Standard drug camptothecin generated 26% of ROS production which confirms higher potential of AgNPs in inducing DNA damage in tumor cells without causing lethality to the healthy cells. Further, the Fluorescence-activated cell sorting (FACS) study using a standard Caspase-3 marker confirms the generation of apoptotic bodies using two different concentrations of MU-AgNPs. At 40 μg, 64% of apoptotic cell death was observed, whereas, using 20 μg, 23% of apoptosis was recorded via fluorescent intensity. Propidium iodide-based Cell cycle study has shown a significant decrease in G0/G1 phase compared to control (88.8%), which further confirmed the apoptotic induction. Matrix metalloproteinases (MMP) studies using JC-1 dye, showed a significant increase in green fluorescence owing to lowered membrane potential, thus ensuring the breakdown of mitochondrial potential compared to untreated and standard drugs. With the obtained results, we are concluding that MU-AgNPs has a tremendous capacity to suppress the ovarian cancer cell proliferation *in vitro* by inducing DNA damage and apoptosis.

## 1 Introduction

Nanoparticles are widely used in industry, cosmetics, biotechnology, and nanomedicine due to their unique features and numerous applications. In recent years, research on metallic nanoparticle applications has become one of the most fascinating fields all around the world (Sagadevan et al., 2020). Among them, biomedical application usage has a vast contribution (Yesilot and Aydin, 2019; Karmous et al., 2020). One of the most widely used and commercialized nanomaterials are silver nanoparticles (AgNPs) gaining popularity due to their broad range of applications in biomedicine. Because of their distinctive qualities, such as photonic and catalytic features, antibacterial, anticancer, and antiangiogenic drugs are all made with AgNPs. They could potentially be utilized in the development of novel and better functional materials (Ghosh, 2019; Sanjay, 2019; Silva et al., 2019). AgNPs are nanoparticles with unique physico-chemical properties ranging from 10 to 100 nm in size. Biological approaches, among the multiple synthetic methods for AgNPs, appear to be simple, rapid, non-toxic, reliable, and green procedures that may produce size and shape that are well defined under ideal conditions for traditional study. Plant-mediated AgNPs are non-toxic, environmentally beneficial, cost-effective, and quickly produced while also acting as reducing, stabilizing, and capping agents. Thus, compared to chemical and physical procedures, the green method of synthesizing AgNPs has several advantages. Green nanoparticles have a high production, solubility, and stability. Metallic nanoparticles that contain medicinal herbs have powerful anti-tumor properties. These metal nanoparticles containing herbs have been utilized to treat ovarian, prostate, oesophageal, stomach and lung cancers, and numerous leukemias in recent years (Shaneza et al., 2018; Mahdavi et al., 2019; Li et al., 2020; Liu et al., 2020).

Ovarian cancer is the world’s second most prevalent gynaecological malignancy, and the fifth most common cancer overall (Momenimovahed et al., 2019). Obesity and overweight, gynaecological surgery, hormone therapy, breast cancer, age, family history, reproductive history, human, talcum powder, and HPV are all variables that increase the risk of ovarian cancer (Ebell et al., 2016). Ovarian cancer started as a single aberrant cell that spread throughout the uterus and the rest of the body (Grossmann et al., 2018). Lethargy, weight loss, nausea, bloating, backpain, early satiety, abdominal pain, constipation, sudden vaginal bleeding, urine frequency, bloating, and dyspnoea are all the signs and symptoms of ovarian cancer. Blood tests, biopsy, laparoscopy, pelvic ultrasound and imaging tests like MRI (magnetic resonance imaging), CT scan (computed tomography), PET scan (positron emission tomography), Chest X-ray are all used to diagnose ovarian cancer (Grossmann et al., 2018). Most cases are discovered after they have progressed to an advanced stage (Vargas, 2014). In women with ovarian cancer, preliminary treatment included surgery followed by platinum-based chemotherapy (Brastasz et al., 2006). Even though majority of women respond to first treatment, chemoresistance develops with time. Cancer recurrence is aided by the great degree of heterogeneity found within ovarian tumors, which is a major feature of the disease, as well as between different ovarian cancer subtypes. Chemotherapy, immunotherapy, and radiation therapy are all used by many clinicians to treat various types of malignancies. Patients with ovarian cancer benefit from a combination of chemotherapy at first, but resistance develops over time (Pokhriyal et al., 2019). Chemotherapeutic medications have a negative impact on the body, so developing an effective chemotherapy treatment using metallic nanoparticles is critical nowadays. Natural product exploitation is one of the most effective strategies for discovering new hits and leads among the numerous tactics (Newman and Cragg, 2012).

Reactive oxygen species (ROS) are the main source of cellular oxidative stress. The regulation of ROS equilibrium is crucial for cellular development, metabolism, and survival (Kim et al., 2016). For tumor formation, ROS can be a two-edged sword (Srinivas et al., 2019). ROS-induced oxidative DNA damage, on the other hand, may cause cell death (Wang et al., 2020). One strategy for tumor cells to achieve this goal is to increase the expression of redox protection proteins, which helps to control intracellular ROS levels (Azzouz et al., 2021). This adaptation would allow the tumor-promoting effects of ROS to be separated from their tumor-suppressive effects (Preya et al., 2017). The increased levels of superoxide radicals fluctuate the mitochondrial transmembrane potential and disrupt the signalling pathway, causing apoptosis and cell death (Fani et al., 2016; Kim et al., 2017). Increased ROS generation damages biological components, including DNA fragmentation, lipid membrane peroxidation, and protein carbonylation. Caspases 3 and 9 are activated by altered mitochondrial membrane potential, resulting in cellular death. It then stimulates a number of downstream signalling pathways, resulting in the creation of apoptotic bodies and cell cycle arrest (Ratan et al., 2020). Cell death in tumor cells is triggered by an increase in ROS, cell cycle arrest, and caspase-3 activation (Kumari et al., 2020). Furthermore, ovarian cancer has been linked to the damaging effects of ROS by reducing the production of antioxidant enzymes (Jiang et al., 2011).

Because of its simplicity and eco-friendliness, plant-mediated biological nanoparticle production has gained popularity in recent years. Plants are a better platform for production of nanoparticles since they are free of hazardous chemicals and contain natural capping agents. Many medicinal plants are used to treat ovarian cancer in traditional medicine, including *Allium sativum*, *Zingiber officinale*, *Dioscorea bulbifera*, *Camellia sinensis*, *Quercus tinctoria*, *Camptotheca acuminate*, *Piper capense*, *Podophyllum peltatum*, *Albizia schimperiana*, *Taxus brevifolia*, *Azadirachta indica*, *Asparagus racemosus*, *Ginkgo biloba*, *Symplocus racemosa*, *Saraca indica*, and *Solanum aculeastrum* (Palanisamy et al., 2021; Aman et al., 2018). Metal nanoparticles produced and manufactured using these plants are expected to have substantially better anti-cancer effects against ovarian cancer cells. Plant-mediated AgNPs have been shown to exhibit anticancer properties in PA-1 (Ovarian cancer cells) (Palle et al., 2020). The G1 phase cell division was delayed by these green synthesized AgNPs, demonstrating that AgNPs can control the cell cycle and limit cell proliferation (Javed and Mashwani, 2020).

*Macrotyloma uniflorum* Linn. (Horsegram), is one of the less well-known grain legume species belonging to family Fabaceae (Singh et al., 2013). In many parts of India, *Macrotyloma uniflorum* grains are commonly consumed as a green vegetable and are believed to be high in protein and have medicinal benefits (Prasad and Singh, 2015). Horse Gram seeds contain bioactive compounds such as phenolic acids and isoflavonones, which play a key role in enhancing antioxidant enzymes in cancer cells thus imparting anticancer properties (Ingle, et al., 2020). Horsegram, is used for asthma, bronchitis, leukoderma, urine discharges, kidney stones, and heart disease, according to traditional medical sources (Marimuthu and Krishnamoorthi, 2013). Green synthesized AgNPs made from a bioactive fraction of *M. uniflorum* was found to have anti-cancer properties (Anjum et al., 2021). Despite the fact that horse Gram is thought to play a key role in modern diets, little research has been done on its cytotoxic effects. *M. uniflorum* seed methanolic extract is used to make silver nanoparticles in this study. The synthesized nanoparticles using *Macrotyloma Uniflorum*, hereafter mentioned as (MU-AgNPs) were examined using a variety of analytical methods. The biological activity was tested against a common ovarian cancer cell line, PA-1, for antioxidant, cytotoxicity, and anticancer properties.

## 2 Materials and methods

### 2.1 Seed collection

*Macrotyloma uniflorum* seeds were obtained from local markets in Tamilnadu, India. Identification and confirmation of these seeds was done by Dr. B. Nagaraj, Dept. of Botany, Sri Venkateswara University, Tirupati, India. The seeds were washed by removing stones and dried in a shady place. They were then pulverised into a fine powder, sieved, labelled, and kept in a zip lock cover for future investigations.

### 2.2 Plant extract preparation

Coarsely powdered seeds of *M. uniflorum* was used for extraction using methanol solvent. 5 g of powder was mixed with 100 ml of methanol in a thimble of soxhlet apparatus and extracted for 6 h at 64°C. The extract was weighed after being evaporated in a rotary flash evaporator at 64°C at 100 rpm and air dried at 25–35°C. The sample was collected and stored in a scintillation tube for later use.

### 2.3 Synthesis of *M. uniflorum* silver nanoparticles

To make up the volume to 5 ml, 2 ml of extracted material was mixed with 3 ml of distilled water. The extract was treated with 10 ml of a 1 mM AgNO_3_ solution, and the tubes were kept in a water bath at 70–80°C for 20 min to allow the reduction reaction to take place. The presence of silver nanoparticles was confirmed by a shift in color from pale yellow to brown in the solution (AgNPs).

### 2.4 Characterization of biosynthesized MU-AgNPs

The UV-Visible spectrophotometer (NanoDrop 8,000 Spectrophotometer) in the 200–600 nm range was used to characterize the biosynthesized AgNPs. The crystalline structure of AgNPs was investigated using X-ray diffraction (XRD) was recorded using the XRD6000 (Shimadzu) in the 2θ range (30–80) (Shimadzu IR). The presence of functional groups in MU-AgNPs was detected using the potassium bromide (KBr) pellet approach on a Shimadzu IR 8400 FTIR (Fourier transform-infrared spectrum) Spectrophotometer (Shimadzu IR) in the 4,000–400 cm^−1^ range. Philips EM208S was used to record transmission electron microscopy (TEM) images to assess the size and shape of AgNPs. The average particle size distribution, hydrodynamic diameter, stability, and polydispersity index (PdI) of MU-AgNPs were determined and measured using a dynamic light scattering (DLS) particle size and a zeta potential analyzer (Horiba Nanopartica SZ-100 Nanoparticle analyzer). The clear nanoparticles solution was used for this analysis after the samples were filtered using 0.2 mm syringe filters, with measurements conducted at the appropriate range.

### 2.5 Antibacterial activity of MU-AgNPs

Anti-bacterial activity was tested against *Escherichia coli*, *Bacillus subtilis*, *Klebsiella pneumonia*, and *Staphylococcus aureus* on nutrient agar plates. Using an L-shaped glass rod, bacterial cultures were evenly dispersed on the surface of the solidified agar media in Petri dishes overnight. 5 mm diameter discs were made using sterile Whatman filter No. 1 paper, and discs were placed equidistantly on each of the inoculated Petri plates with the help of sterile forceps. By using a micropipette and sterile micropipette tips, biosynthesized silver nanoparticles at various concentrations ranging from10–40 μg/ml were added to the discs. The centre of the plate was filled with 30 µl of crude sample disc. Antibiotic disc plates were made by placing levofloxacin antibiotic discs in the centre of the agar plates as a positive control and incubating them overnight or for 12–16 h at 37°C. The clear zone appeared after incubation was measured as an inhibitory zone (Cos et al., 2006).

### 2.6 DPPH activity of MU-AgNPs

The free radical scavenging assay was determined with the 2,2-Diphenyl-1-picrylhydrazyl radical (DPPH) (Tahvilian et al., 2019). Different concentrations of green synthesized nano and crude samples were taken, 100–500 µg/ml, and made the final volume of 2 ml by adding methanol. To each tube, 1 ml of DPPH was added. The absorbance of the mixtures was measured at 570 nm after 30 min of incubation in dark. 3 ml of DPPH was used as a control. MU-AgNPs and the plant crude extracts were measured and expressed as percentage of inhibition (%).

$$\text{Inhibition }\left(\text{\%}\right)\;=\;\frac{\left[\text{Abs}\right]\text{ control }-\;\left[\text{Abs}\right]\text{ sample}}{\left[\text{Abs}\right]\text{ control}}X100$$ (1)

### 2.7 Anti-cancer activity of MU-AgNPs

The antitumor activity of MU-AgNPs was assessed *in vitro* using the MTT (3-(4,5-dimethylthiazol-2-yl)-2,5-diphenyltetrazolium bromide) method against PA-1 cells (You et al., 2012). In a nutshell, cells were grown and maintained in DMEM (Dulbecco’s modified Eagle’s medium) pH 7.4 with 10% foetal bovine serum (FBS), glutamine (2 mM), penicillin—streptomycin (100 mg/ml). Cells were seeded at a density of 0.1 million in a 96 well plates. After 24 h, the cells were treated with different concentrations of MU-AgNPs ranging from 50–300 μg/ml. After 24 h treatment, each well was filled with 15 µl of MTT (5 mg/ml) in phosphate buffered saline (PBS) and incubated for 4 h at 37°C. The MTT medium was then flicked off, and the formazan crystals that had formed were dissolved in 100 µl of dissolving solution before being measured at 570 nm with a microplate reader. Data was plotted by calculating the viability percentage.

$$\frac{\left[\text{Abs}\right]\text{ Test}}{\left[\text{Abs}\right]\text{control}}\text{ x }100\;=\text{ Percentage of cell viability}$$ (2)

$$\frac{100-\text{ Abs }\left(\text{sample}\right)}{\text{Abs }\left(\text{control}\right)}\text{ x }100\;=\text{ Percentage of cell inhibition}$$ (3)

### 2.8 Flow cytometric analysis using propidium iodide

PA-1 cells were planted at a density of 1*10^5^ cells per well in a 6-well plate and cultured for 24 h at 37°C in a CO_2_ incubator. The cells were treated the next day with the drug, MU-AgNPs, based on the IC_50_ value. The cell control received no treatment. The treatment was administered for two different periods of time, namely 24 and 48 h. Cells were trypsinized and centrifuged at 8,000 rpm for 5 min and the pellet was collected. The cells were rinsed in cold PBS and 1 ml of 70% chilled ethanol was added by vortexing. Ethanol was removed by centrifuging at 2000 rpm for 15 min at 4°C followed by a PBS wash. 50 µl of RNase A was added to the pellet and incubated for 30 min at 37°C. The cells were given 450 µl of PI solution. The cell cycle profile for 10,000 events was obtained using the Fluorescence-activated cell sorting (FACS) calibrator.

### 2.9 Annexin V/PI apoptotic assay

PA-1 control, untreated and treated (AgNPs) cells were seeded at a density of 3*10^5^ cells/well in 6-well plates, incubated overnight, and then treated with 5 µM of camptothecin or the control (DMSO). Cells were trypsinized, washed once with PBS, and suspended in 1 × binding buffer after 48 h of treatment. After that, each sample was incubated for 15 min at room temperature in the dark with annexin V-FITC (5 μl) and PI (5 μl). Flow cytometry was used to examine the cells (BD Biosciences, San Jose, CA, United States). After 48 h of treatment, cells were trypsinized, washed once with PBS, and then suspended in 1× binding buffer. Annexin V-FITC (5 μl) and PI (5 μl) were then added to each sample, followed by incubation for 15 min at room temperature in the dark. Cells were analyzed using flow cytometry (BD Biosciences, San Jose, CA, United States).

### 2.10 Cell cycle analysis

PA-1 cells were seeded in six well plate and incubated overnight. After 24 h, cells were treated for 24 h with 40 μg of MU-AgNP extract and camptothecin (5 µM). After trypsinization, the pellet was collected, reconstituted in cold ethanol (70%), and frozen for an hour to improve the staining capacity. The cells were washed twice with cold PBS and centrifuged for 5 min at 3,000 rpm. In 0.5 ml of PI/RNase staining buffer, the pellet was resuspended and incubated at room temperature in the dark for 30 min. A FACS Calibur flow cytometer was used to determine the cell cycle distribution (Becton Dickinson, United States). A total of 10,000 gating occurrences were recorded.

### 2.11 Mitochondrial membrane potential (ΔΨm)

Changes in mitochondrial membrane potential were determined by flow cytometry using the fluorescent dye 3,3′-dihexyloxacarbocyanine iodide (DiOC6) to understand the role mitochondria play in PA-1 cell death after treatment with MU-AgNPs (Sigma). In 6-well plates, PK-1 cells (3,104 cells/well) were planted. The cells were washed with PBS and resuspended in 10 nM DiOC6 after being incubated with camptothecin (5 µM) or DMSO for 24 and 48 h. The cells were flow cytometrically examined immediately after 30 min of incubation at 37°C.

### 2.12 Caspase-3 and Caspase-7 activity

The activity of caspase-3 and 7 was evaluated *via* flow cytometry according to the method described by Moraes et al. (2013), with minor modifications. Cells were seeded in 24-well plates (105 cells/ml) and grown in media containing 10% FBS for 24 h after being treated with the AECL or AECR IC_50_. The cells were centrifuged, washed, and fixed in 4% paraformaldehyde in PBS for 30 min after treatment. The cells were then washed in PBS containing 0.1 M glycine, permeabilized with 0.01% saponin for 30 min, then blocked for 30 min at room temperature in PBS containing 1% bovine serum albumin (BSA). The cells were then incubated in the dark at room temperature for 40 min with an anti-active caspase-3 monoclonal antibody labelled with FITC (BD Pharmingen, San Diego, CA, United States). Fluorescence was analysed using Cell Quest software in a FACS Calibur flow cytometer (Becton Dickinson, United States) after incubation (10,000 events were collected per sample).

### 2.13 Reactive oxygen species study

In a growth medium, 0.1 million cells were sown in a 6-well plate and incubated for 24 h. Cells were treated for 24 h with MU-AgNPs (20 and 40 μg/ml) and 5 µM Camptothecin. Cells were trypsinized and washed three times with PBS (pH 7) after 24 h of treatment. The supernatant was discarded after centrifuging the cells. The H2DCFDA reagent (2 M, 1 ml) was then applied to the collected pellets, and the cells were incubated at room temperature in the dark for 1 h. The pellet was washed with Dulbecco’s phosphate-buffered saline (DPBS), pH 7.4. The fluorescence intensity (FL1-H) of each cell pellet was then measured using FACS equipment (write the model type). A total of 10,000 gate events were measured for each analysis.

### 2.14 Statistical analysis

Data were expressed as mean ± SEM in triplicate. Statistical analysis was performed using one-way ANOVA (SPSS version 17).

## 3 Results

### 3.1 Characterization studies

#### 3.1.1 Green synthesis of silver nanoparticles

**FIGURE 1:**
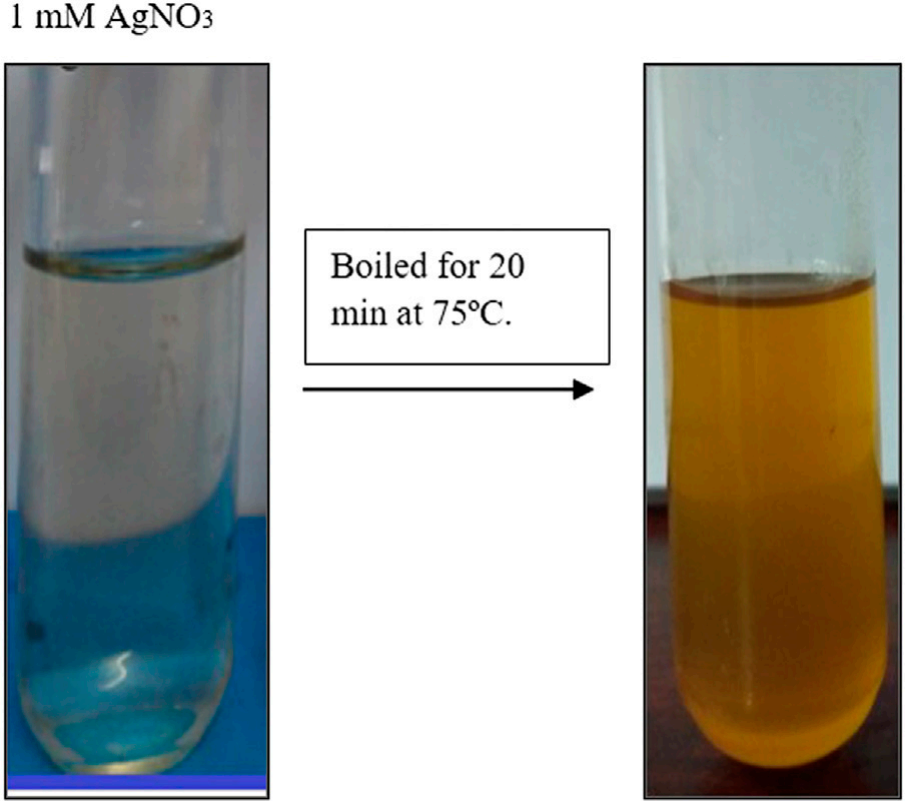
Represents the reduction of *Macrotyloma uniflorum* silver nanopracticles. Change colour to light brown confirms the synthesis.

The generation of AgNPs was confirmed by the change of color from transparent to dark brown by reduction process. Enhancement of the color is a confirmation for AgNP production. This color change was occurred due to the presence of several bioactive compounds such as tannins, flavonoids, terpenoids, phenols, amines etc. Figure 1 represents the change of colour which confirms the AgNP synthesis.

#### 3.1.2 UV-vis spectrophotometer

**FIGURE 2:**
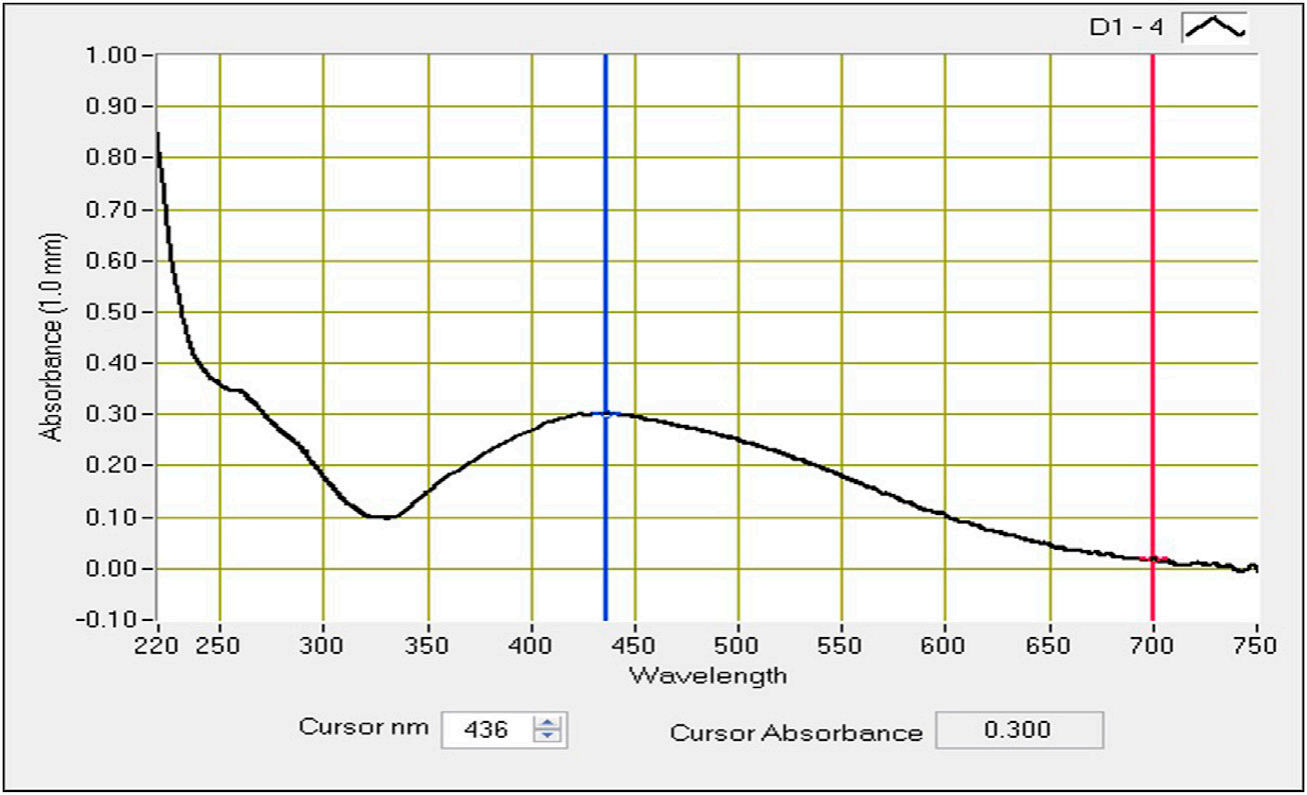
UV-visible absorbtion spectra of AgNPs synthesized from *Macrotyloma uniflorum* seed extract with 1 mM silver nitrate.

Spectrophotometry studies confirmed the distant surface plasmon resonance bands at a peak of 436 nm (Figure 2) in *Macrotyloma uniflorum* seed extracts. Similar results were obtained in Vidhu et al. (2011).

#### 3.1.3 Particle size determination

**FIGURE 3:**
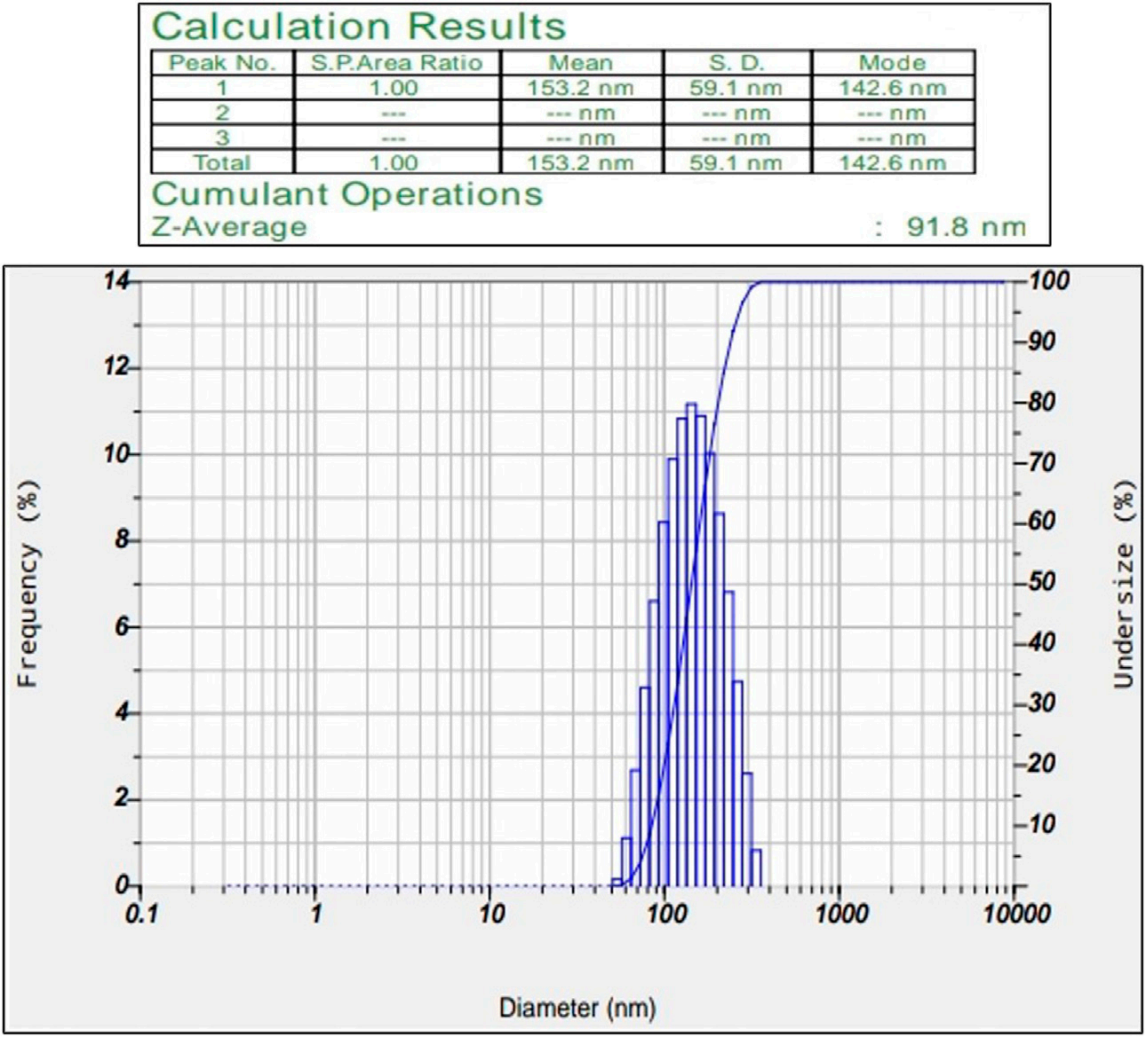
Show the average Z-value particle size of synthesized silver nanoparticle.

Synthesized silver nanoparticles range from a size of 50–100 nm. Obtained particles in our study have an average diameter of 91.8 nm confirms their identity as AgNPs (Figure 3). Nindawat and Agrawal (2019) studies confirmed the silver nanoparticle size can be either lower (20–60 nm) or medium (200–500 nm). This helps us in confirming the size of the synthesized nanoparticles.

#### 3.1.4 Zeta potential

**FIGURE 4:**
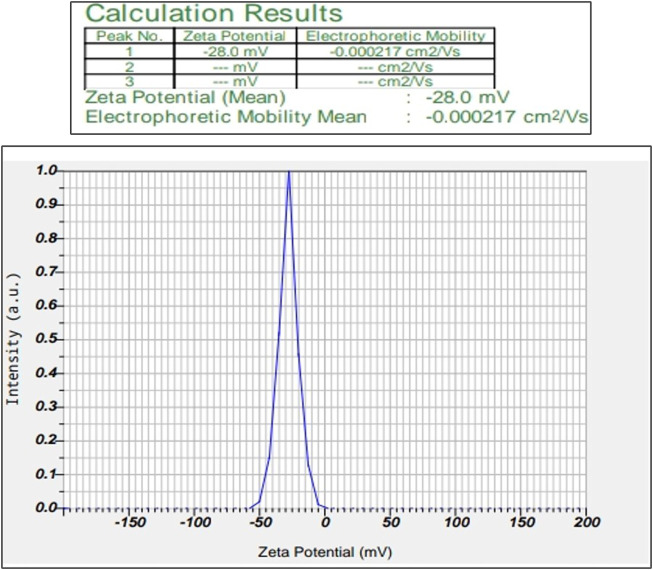
Shows Zeta potential of silver nanoparticles *Macrotyloma uniflorum* seed extracts.

This study helps us to reveal the possessed charge of the synthesized MU-AgNPs. Electrostatic charge Zeta potential obtained by these particles is −28.0 mV (Figure 4). The negative charge helps the AgNPs from aggregate formation due to the repulsion among themselves. Many studies have confirmed the negative charge in various AgNP synthesized plant extracts.

#### 3.1.5 XRD studies

**FIGURE 5:**
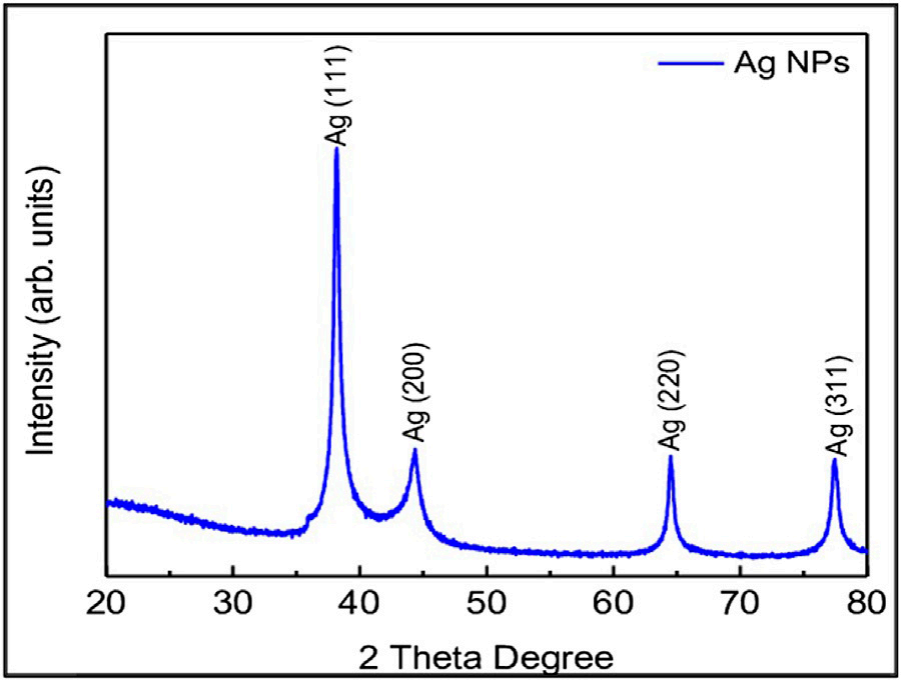
The peaks showing X-RAY Diffraction of AgNPs.

X-ray diffraction technique (XRD, D/Max 2005; Rigaku) was used to investigate the crystal phase structure of the AgNPs, synthesized using leaf extracts. Crystal phase of AgNPs is determined using XRD. AgNPs, which was synthesized using HG extract (Figure 1) has shown four distinct peaks at 2θ = 38.17, 44.36, 64.52, and 77.46 were assigned to (111, 200, 220, and 311) planes of face centered cubic (FCC) structure of metallic silver (JCPDS 04–0,783) with Fm3m planetary group symmetry (Figure 5). Scherrer’s equation is used to calculate the mean crystallite diameter (MCD) of as synthesized nanoparticles, and the size is found to be ≈20–30 nm of AgNPs.

#### 3.1.6 FTIR studies

**FIGURE 6:**
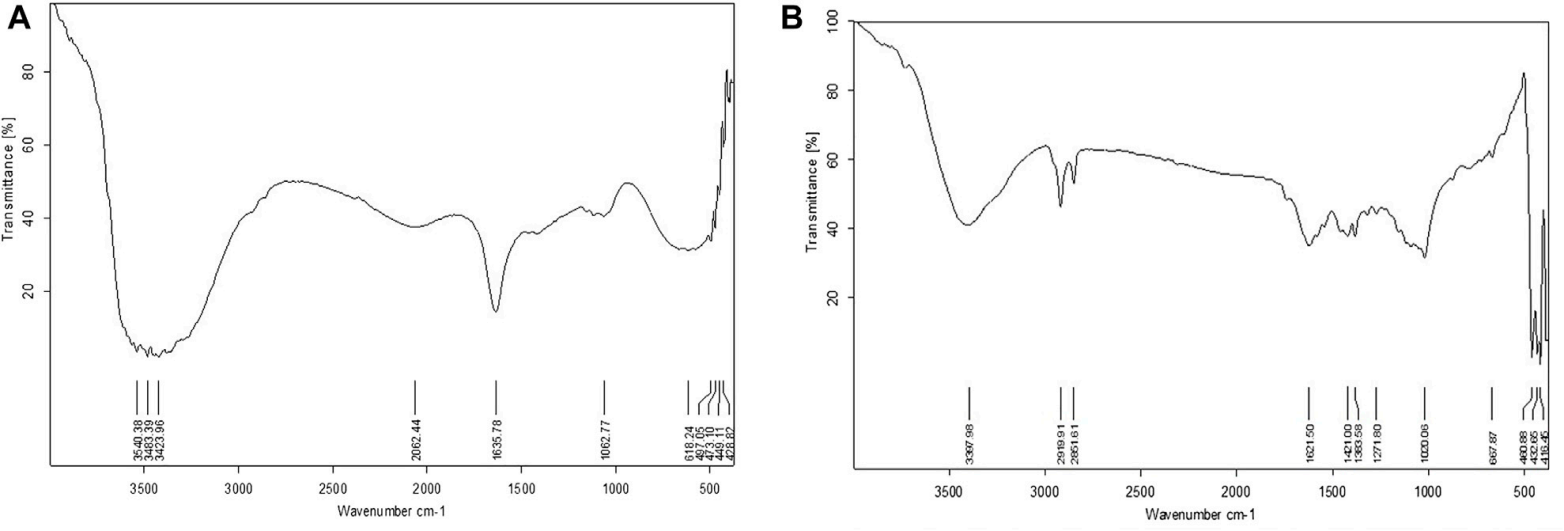
FTIR analysis of crude **(A)** and AgNP **(B)** samples of *Macrotyloma uniflorum* seed extracts.

FTIR spectrum of methanolic seed extract showed significant peaks at 3,540.38, 3,483.39, 3,423.96, 1,635.78, 2062.44, 1,062.77, 618.24, 497.05, 473.10, 449.11, and 428.82 cm^−1^ which corresponds to the presence of R-COOH, N-H, C=O, C≡C, Ethers, C-Br, C-I stretch, respectively. The nano extract of seed showed significant peaks at 3,397.98, 1,621.50, 2,919.91, 2,851.61, 1,421.00, 1,383.58, 1,271.80, 1,020.06, 667.87, 480.88, 432.85, and 416.45 cm^−1^ which corresponds to the presence of O-H, N-H, C=O, C-H, Ethers, C-Br, C-I respectively. (Figure 6).

#### 3.1.7 TEM analysis

**FIGURE 7:**
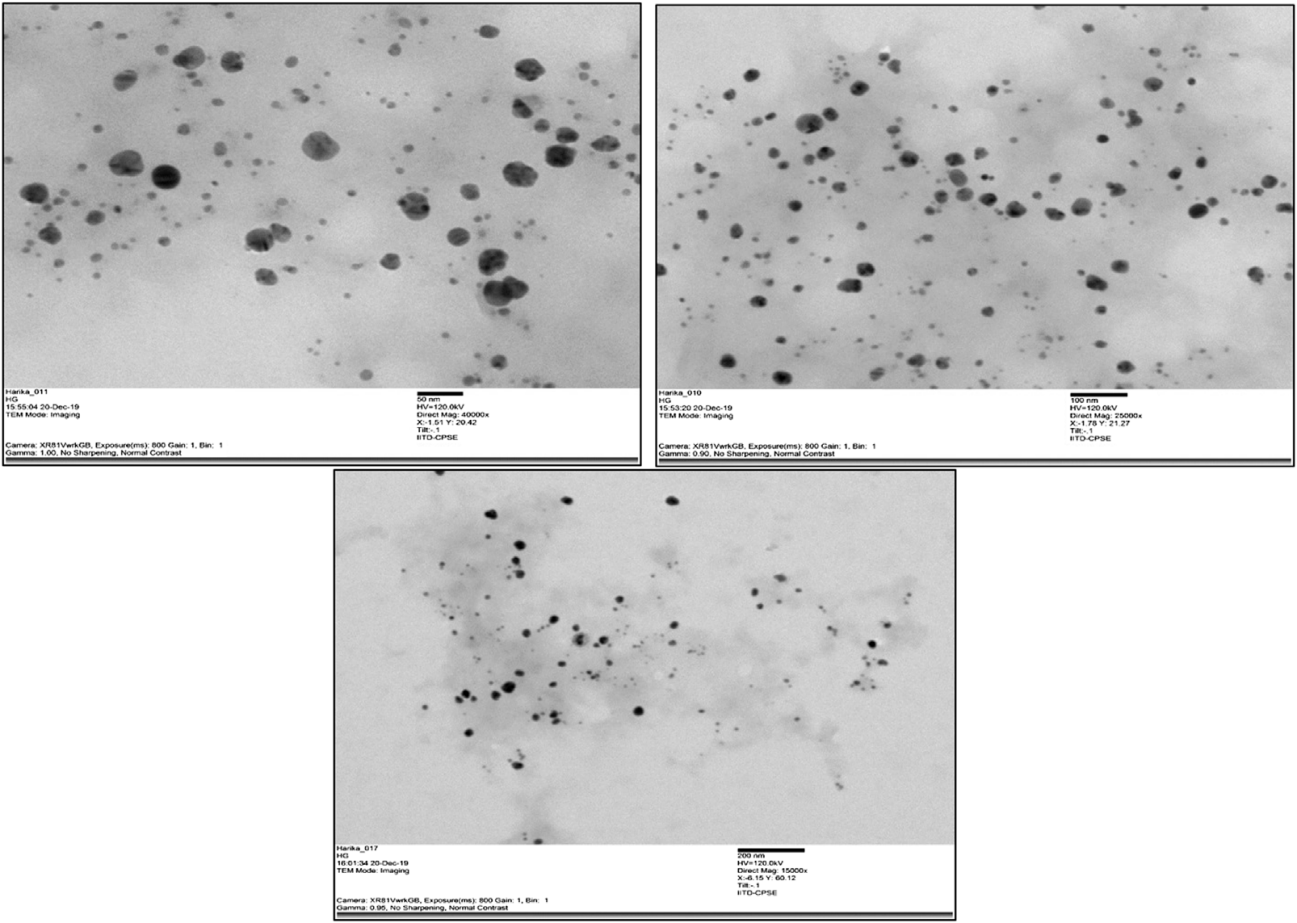
TEM images of synthesized nanoparticles from *Macrotyloma uniflorum* at 50, 100, and 200 nm respectively.

The TEM images of the silver nanoparticles biosynthesized from *Macrotyloma uniflorum* shows spherical and oval shapes at 50, 100, and 200 nm. (Figure 7).

### 3.2 Anti-bacterial activity

**FIGURE 8:**
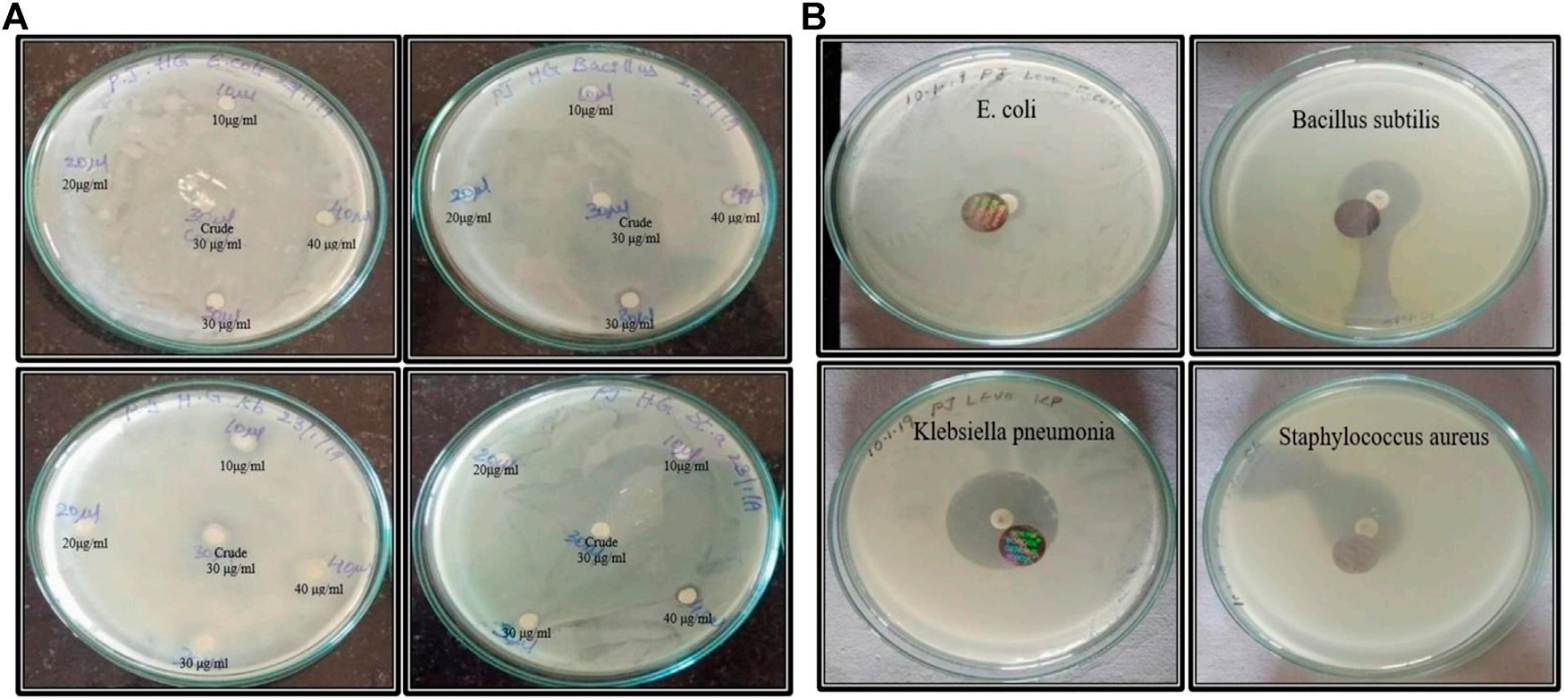
**(A)** Anti-microbial activity of both crude and nano synthesized samples. **(B)**Anti-microbial activity of board-spectrum Levofloxacin antibiotic with *E. coli*, *Bacillus subtilis*, *klebsiella pnemoniae*, and *staphylococcus aureus* respectively.

By using the disc diffusion technique on nutrient agar plates, the obtained results *S. aureus* has shown a range of inhibition zone compared to other microbes. Hence, we have seen a minute anti-bacterial activity using MU-AgNPs. Figures 8A,B shows the zone of inhibitions between plant extracts and levofloxacin. (Supplementary Table S1).

### 3.3 Free radicle scavenging assay

**FIGURE 9:**
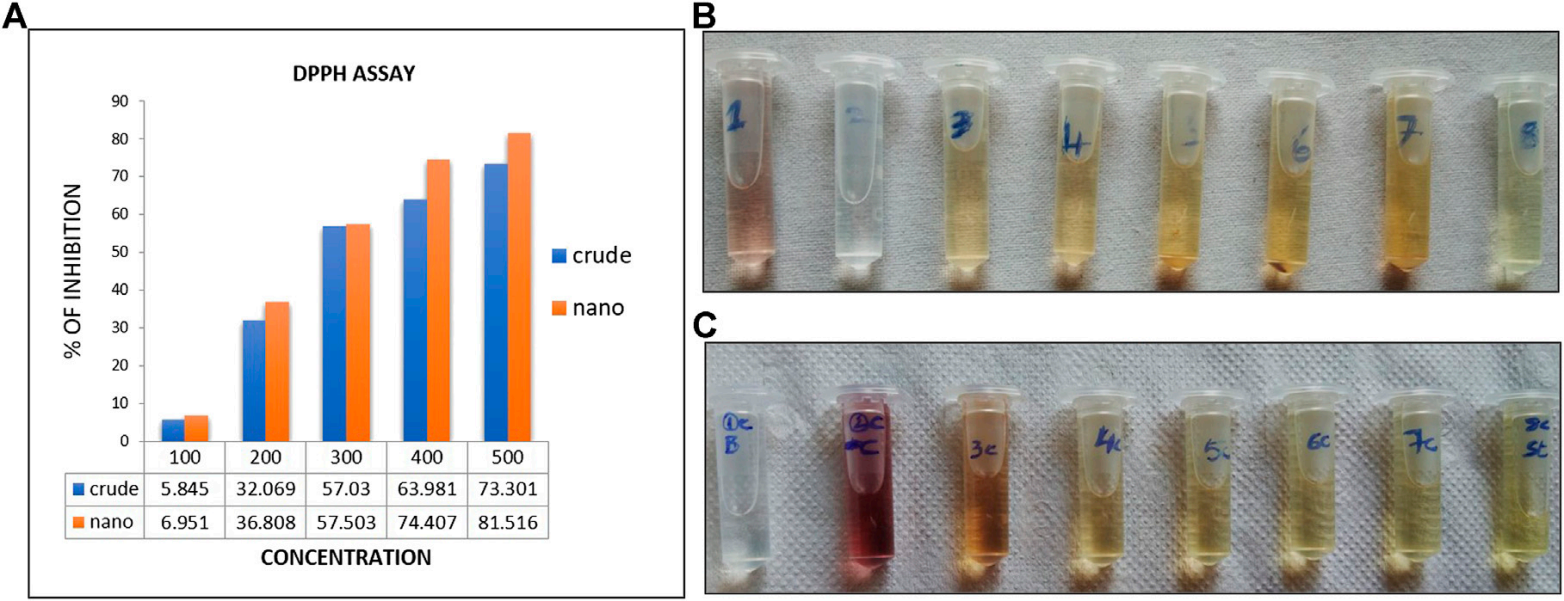
**(A)**-indicates the % of free radicle inhibtion through DPPH activity. **(B,C)** indicates the colur change in crude extracts **(B)**and AgNP **(C)** synthesized extracts.

Antioxidant activity of crude and AgNPs synthesized from *Macrotyloma uniflorum* plant was checked by DPPH free radical scavenging assay. This method is dependent on the reduction of DPPH radical to the non-radical form DPPH-H in the presence of hydrogen donating antioxidant. The % of inhibition of crude and AgNPs were represented in the Figure 9A. The % of inhibition of AgNPs was increased with increasing concentration and the highest percentage was observed at highest concentration of 500 μg/ml used in this assay and was found to be 76.08%. DPPH assay is an easy, rapid, and sensitive way to survey the antioxidant activity of a specific compound of AgNPs. (Figures 9A–C).

### 3.4 Cytotoxicity studies

**FIGURE 10:**
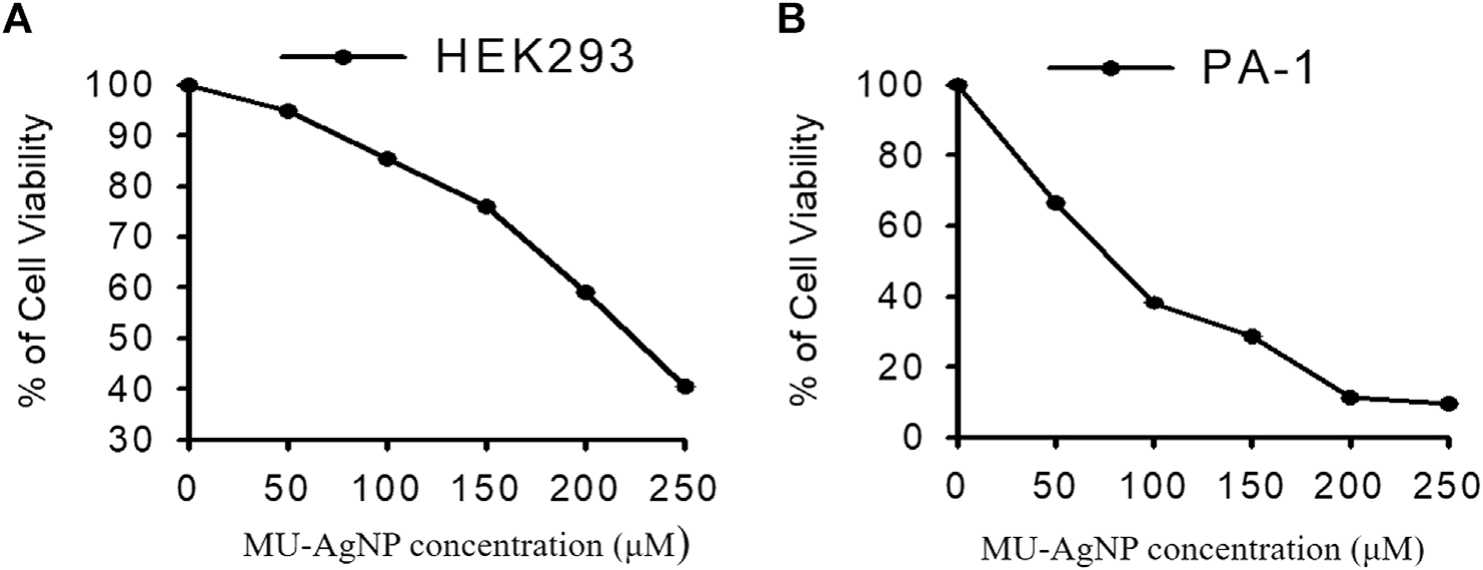
Represents the anti cancer activity of Horse Gram AgNP extracts in both **(A)**-HEK293 and **(B)**-PA-1 cells by MTT assay method.

Cytotoxicity studies were performed in Ovarian cancer cell line PA-1 by using MU-AgNP samples at different concentrations (0–250 µg) (Figure 10A). At 50 µg concentration, 66.6% of viability was observed and at 250 μg, 9% viability was observed which shows that these MU-AgNPs have a higher efficiency to inhibit the cancer cell growth compared with the control cell line HEK293 (Figure 10B) which has a viability around 40% at the highest concentration.

### 3.5 MU-AgNP promotes apoptosis

**FIGURE 11:**
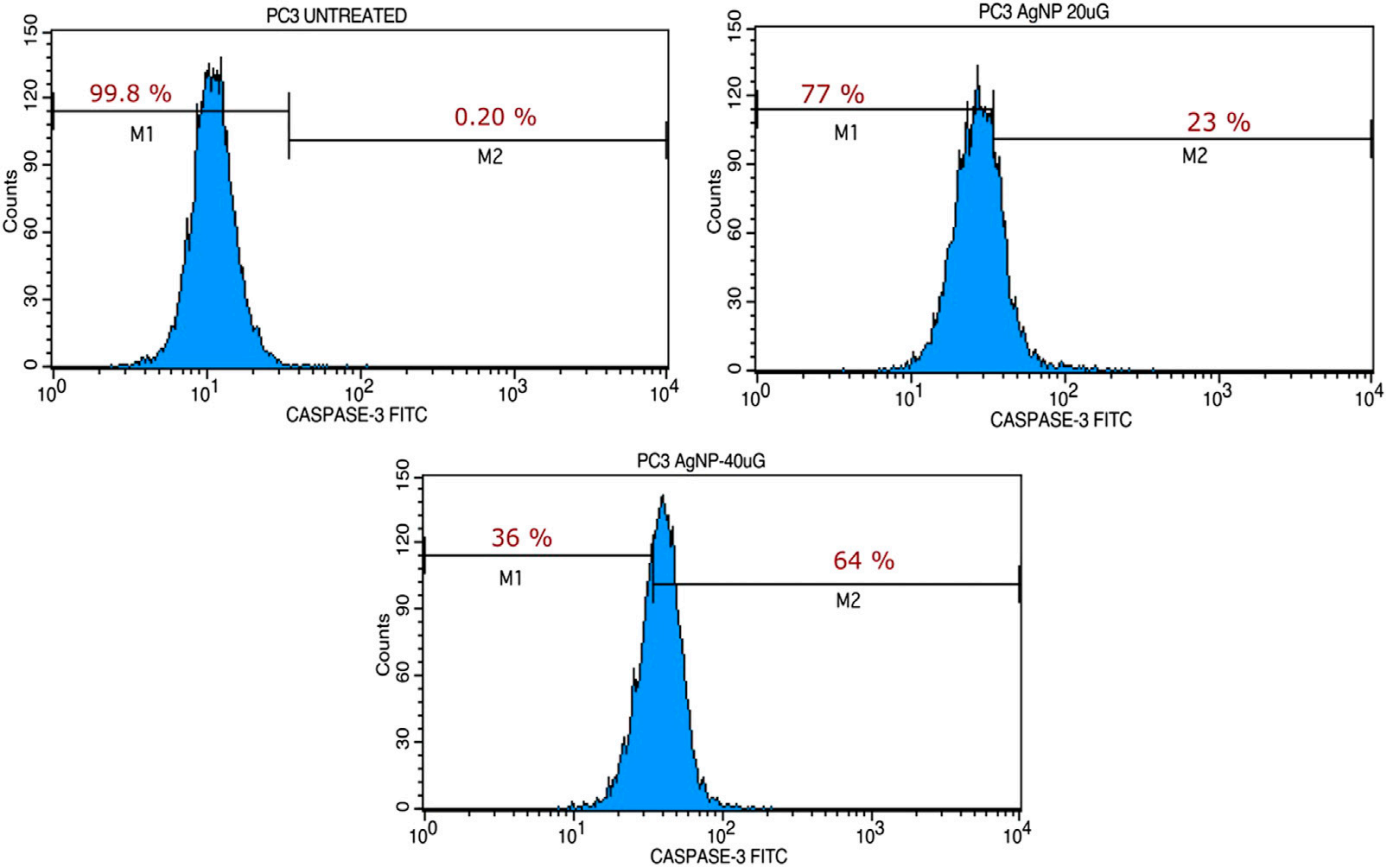
Shows the capase the activity at 20 and 40 µg concentration treated with MU-AgNPs.

Our studies indicated the ability of MU-AgNP to influence cell viability. Caspase-3 marker assay is a standard analysis carried out using FACS to understand the potential of a compound as an apoptotic agent. Hence, the Caspase-3 was designed using two different concentrations of Mu-AgNP obtained from Annexin V assay, to evaluate its apoptotic potential in PA-1 cells. FACS analysis of the Caspase-3-FITC stained cells revealed efficient concentration dependent initiation of apoptosis by MU-AgNP. The results showed a significant, MU-AgNP concentration dependent increase in PA-1 cells apoptotic population compared to the control untreated group (Figure 11). At 20 µg & 40 µg MU-AgNP exerts 23 and 64% of apoptotic cell death as per the caspase-3 fluorescent intensity increases in treated cells. Notably the potential of MU-AgNP to selectively to induce apoptosis in a concentration dependent manner is an ideal property for cancer therapy related drugs. The results obtained from FACS were further confirmed by cell cycle analysis and CLSM imaging of the PA-1 cells using other related studies.

### 3.6 Cell cycle analysis MU-AgNP causes DNA damage and cell cycle arrest

**FIGURE 12:**
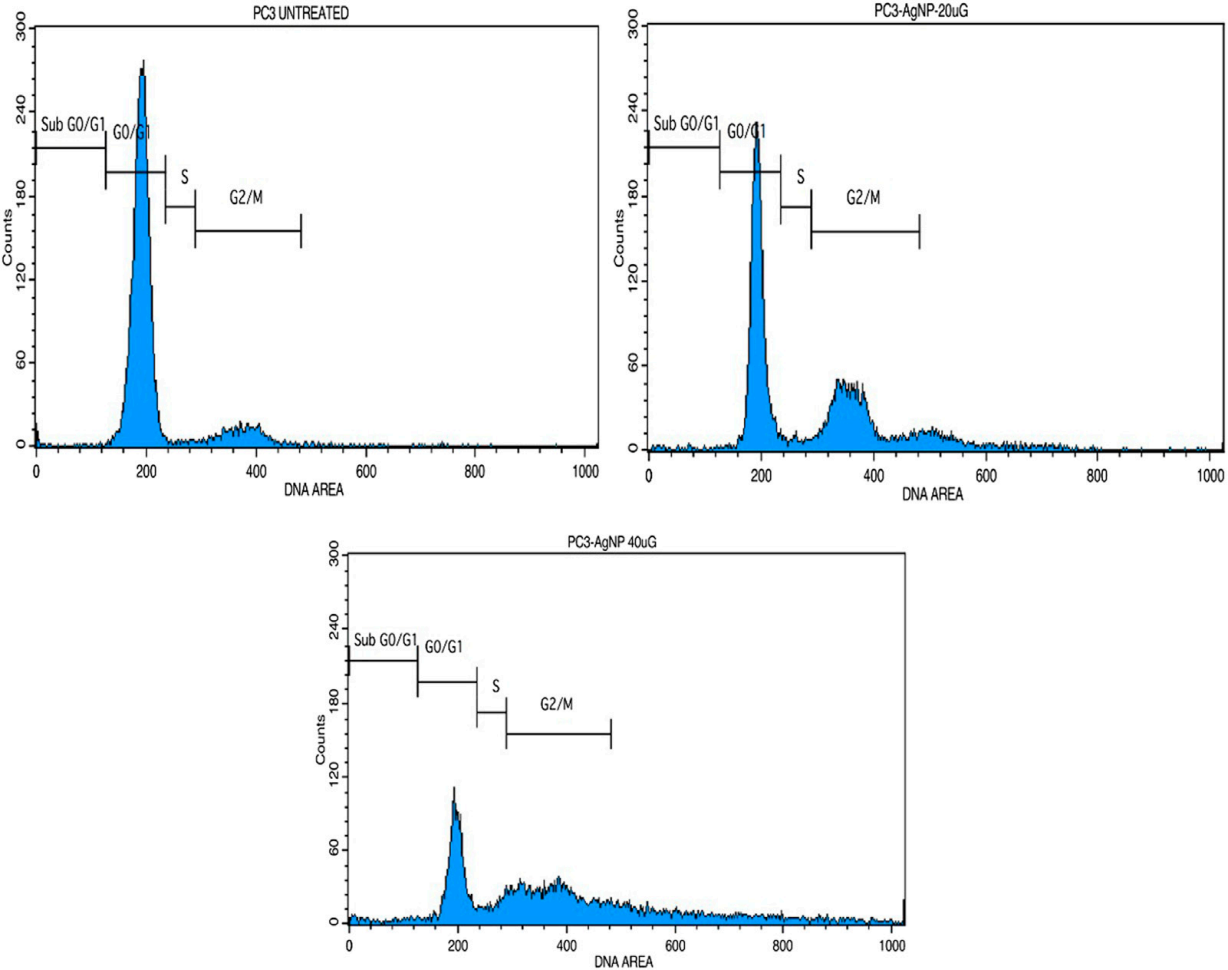
Shows the cell cycle analysis of MU-AgNPs treated with control and 20 and 40 µg concentration.

Based on the earlier results it would be of interest to study the influence of MU-AgNP on different phases of PA-1 cell cycle. One of the properties of an effective anticancer drug is to selectively target a specific phase in the life cycle of the cancer cell. PI based cell cycle analysis was carried out using FACS to identify the influence of MU-AgNP on different phases of the PA-1 cell cycle (Figures 12A–C). Results from the cell cycle analysis showed increase in G_2_/M phase population with a concomitant decrease of cells in the SubG_0_/G_1_ phase (Figures 12B,C). In contrast there is a considerable increase in SubG_0_/G_1_ which is characterized by DNA damage and a feature of apoptotic cells. Cells treated with MU-AgNP showed a significant decrease in G_0_/G_1_ phase (∼28.8 & 54%) compared to the control group (88.8%). Whereas the S phase cell population showed significant increasing trend compared to the control group. The cell cycle analysis results further confirmed the potential of MU-AgNP as an anticancer agent with ability to induce apoptosis in PA-1 cell line.

### 3.7 PC3 AgNP induces mitochondrial membrane damage

**FIGURE 13:**
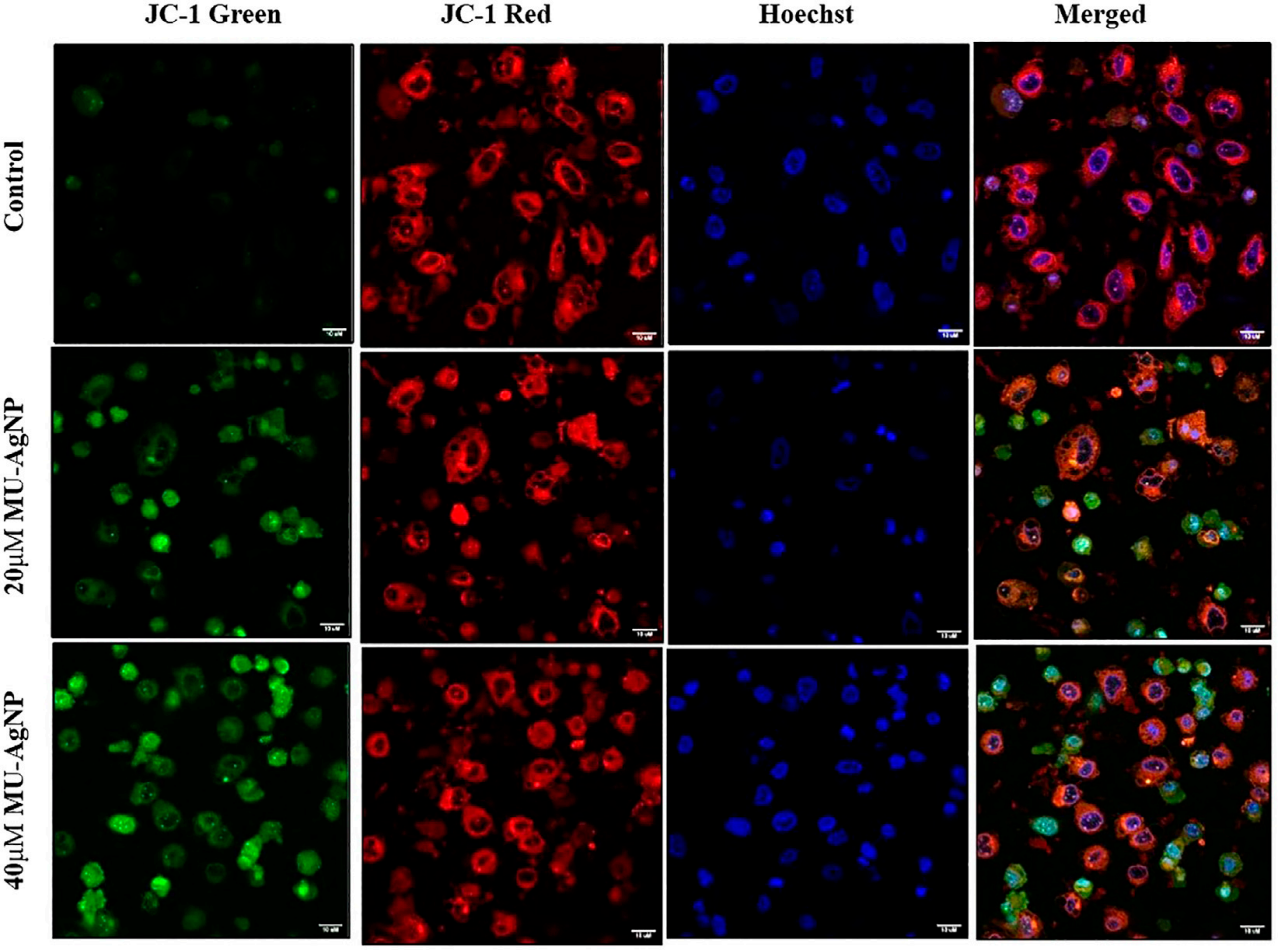
Shows the mitochondrial membrane potential activity of MU -AgNPs treated with control and 20 and 40 µM Concentrations. Increase in JC-1 green staining in the treated samples confirms the break down of mitochondria resulted due to apoptosis.

Apoptosis is often associated with the induction of cellular oxidative stress and altered membrane function in organelles such as mitochondria. The level of altered membrane function or membrane damages has been shown to have strong correlation with the induction of apoptotic pathway in cells. This mode of apoptotic induction is one of the key features for an anticancer agent. Earlier assay results along with FACS based Caspase-3 assays showed strong ability of MU-AgNP to reduce cell viability of PA-1 cells and induce apoptosis. Hence, the influence of these MU-AgNP on Mitochondrial membrane damages of the PA-cells would be of interest to understand whether the apoptotic pathway of the PA-1 cell is influenced *via* membrane damage. The potential-dependent alteration of membrane potential, treated with MU-AgNP was analyzed using CLSM with the aid of JC-1 dye, the results showed that in comparison with untreated, treated cells showed Significant increase in green fluorescence owing to lowered membrane potential. Thus, the increase in green fluorescence intensity in MU-AgNPs treated cells indicates break-down of the mitochondrial membrane potential in comparison with untreated, which indicates drug induced apoptosis activity. The images concurred with the results observed in FACS indicating that MU-AgNP might be playing a role by altering the mitochondrial membrane potential further leading to apoptosis. (Figure 13).

### 3.8 Reactive oxygen species generation

**FIGURE 14:**
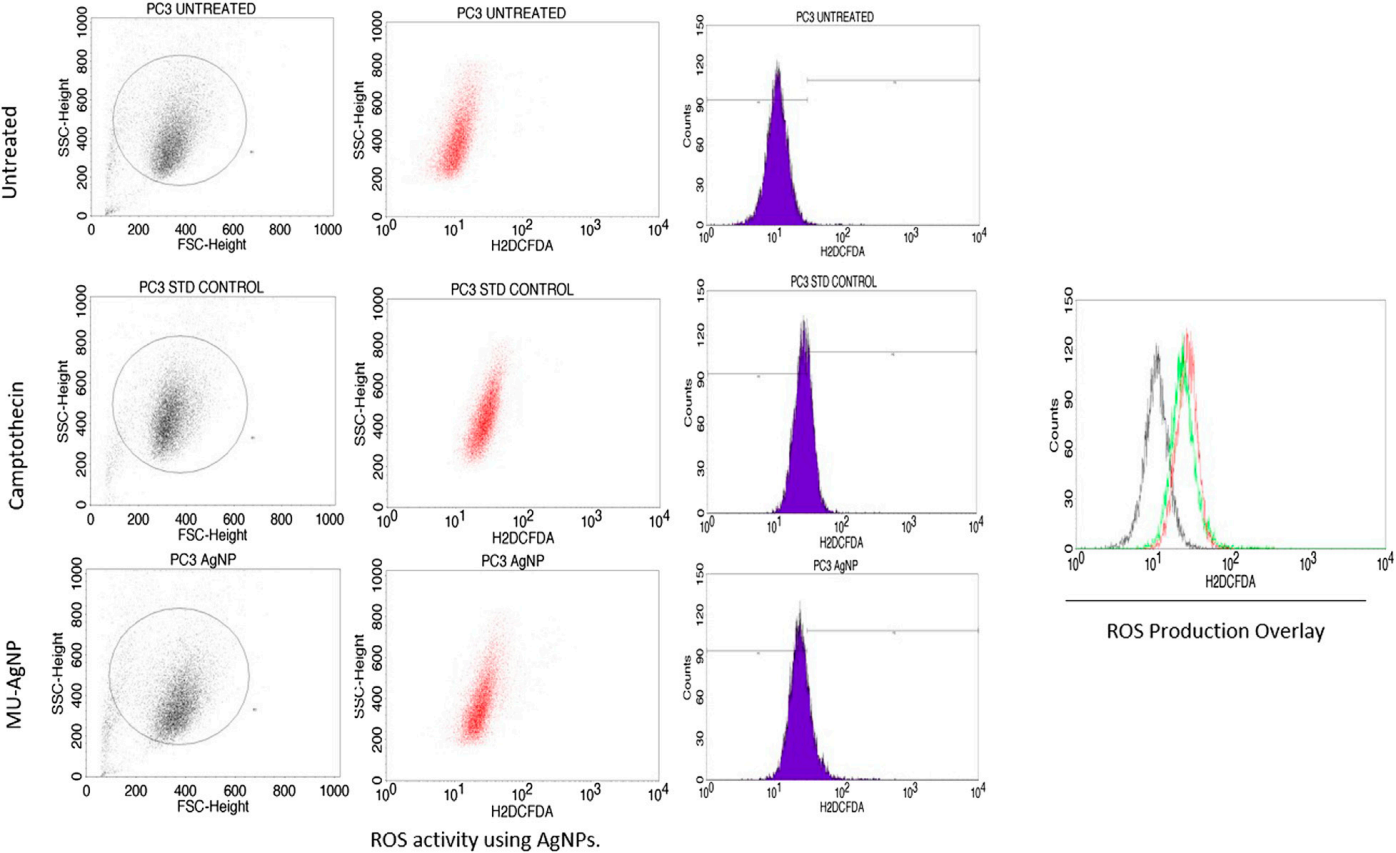
Shows the ROS generation in control, standard and AgNP treated samples.

Several reports have shown that Reactive oxygen species (ROS) is an indication of DNA damage/apoptosis mechanism (Mitra et al., 2019; Ebrahimzadeh et al., 2018). NavaneethaKrishnan et al. (2019) has reported that polyphenols which are a part in phytochemical extracts of a plant are the key compounds for ROS induced DNA damage. MU-AgNP particles has ROS production of 21.3% at 40 µg concentration whereas untreated ones have a percentage of 0.45%. Standard drug camptothecin generated 26% of ROS. Zeta potential studies has shown the negative surface charge of the MU-AgNP which is not the prime factor for both the cytotoxicity and ROS generation. (Cameron et al., 2018). Several studies on cancer using phytochemical extracts from plants and their parts has shown ROS activity as a sign of DNA damage in cancer cells compared to the controls. Ahn and Park (2020) studies have shown that AgNPs has a capacity to generate ROS and thus can cause damage to the DNA in A549 lung cancer cells. Considering this, we reported that ROS production using AgNPs derived from *Macrotyloma uniflorum* seeds have a capacity to generate ROS by damaging the DNA in Ovarian cancer cells along with the standard drug camptothecin. (Figure 14).

## 4 Discussion

Nano based medicine has gained a huge popularity in past decades because of fewer toxicity. Organic synthesis method enhanced its therapeutic activity and hence could be effective to treat against various ailments. In a recent review by Hembram et al. (2018), choosing AgNPs over AuNPs has been highlighted at *in vivo* level and can be supported by the reason of choosing Eco-friendly synthesis of AgNPs which has gained a vast recognition over globe not only because of their effectivity but also due to the efficacy (Srivastava et al., 2018). Utilising the organic plant extracts in treating diseases specifically cancer is highly required considering no damage to the healthy cells which are surrounding in the tumor microenvironment (Srivastava et al., 2018; Singh et al., 2021).

Considering the above, our research mainly focussed on poor man’s legume *Macrotyloma uniflorum* because of its wider availability in the Asian countries. AgNP production was confirmed by analysing the wavelength spectrum from 300–700 nm and observed the wider SPR bands. Macrotyloma seed extracts has showcased a characteristic peak at 436 nm using AgNO_3_ mixture, which likely confirms green synthesis of AgNPs. These results are quiet similar from the work done by Vidhu et al. (2011). Particle size of the silver nano particles plays an important role in the therapeutic strategies. The size of the nanoparticle determines whether the AgNP tagged compound has a capacity to penetrate through the cell membrane and bind to the intra cellular compartments. *M. uniflorum* seed extracts has shown an average size of 91.8 nm which falls into AgNP category. Zeta potential studies revealed the negative charge of the synthesized nano particles which shows the repulsion character among themselves by avoiding the aggregate formation. Crystal studies were determined by XRD, which is an advanced technique to study the crystal nature of MU-AgNPs. TEM analysis has confirmed the spherical structure of nanoparticles. However, our data has not shown significant anti-bacterial activity.

*In vitro* assays have showed significant results which shows similarity with the early reports. MTT assay has showed a significant result in the viability of PA-1 cells compared to the control cell line which confirms that MU-AgNPS have a capacity to inhibit the tumor cell growth (Palle et al., 2020). Further FACS analysis have shown the tremendous in G2/M phase and a concomitant decrease in subG0/G1 phase confirms the presence of apoptotic cells. Further, caspase 3 study by FACS has confirmed the generation of apoptotic cells using different concentration of MU-AgNPs at 23 and 64%. This confirms that MU-AgNPs can cause the tumor cells to death through apoptotic pathway. Our data matches with the other reports who has used different plant AgNP extracts (Bordoni et al., 2021). Finally, mitochondrial membrane potential studies confirmed the reduction of cell viability through apoptosis process, thus staining with JC-1 dye confirmed that MU-AgNPs treated group has shown high intensity thus confers the breakdown of mitochondrial membrane and results in lower membrane potential.

## 5 Conclusion

We are concluding our finding by saying that MU-AgNPs can be a promising treatment regimen to control the proliferation of Cancer cells, particularly Ovarian cancer. Further studies are highly recommended to see whether they have the similar capability to control the growth of resistant tumors and combinational drug treating of MU-AgNPs alongside primary treatment regimens can be highly appreciated. Studies related to cancer cell signaling pathways are much needed to target specific genes.

## Data Availability

The original contributions presented in the study are included in the article/Supplementary Material, further inquiries can be directed to the corresponding authors.
