# Supplementary material for: 2-Dimensional in vitro culture assessment of ovarian cancer cell line using cost effective silver nanoparticles from Macrotyloma uniflorum seed extracts
Source: Front Bioeng Biotechnol. 2022 Aug 16;10:978846. doi: 10.3389/fbioe.2022.978846 (PMC9425338; doi:10.3389/fbioe.2022.978846)
Supplement: Supplementary file 1 [file Table1.DOCX]

**Table 1. The range of zone of inhibition of crude and nano samples for four different bacterial strains**

| **Inhibition Zone (mm)** | | | | |
| --- | --- | --- | --- | --- |
|  | **E.coli** | **B. subtilis** | **S. aureus** | **K. pneumoniae** |
| **Levofloxin** | **10** | **26** | **22** | **34** |
| **10 μg/ml** | **2.4** | **4** | **3** | **5** |
| **20 μg/ml** | **2** | **5** | **5** | **6** |
| **30 μg/ml** | **2.5** | **6** | **8** | **7** |
| **40 μg/ml** | **3** | **7** | **10** | **7** |
